# Manual therapy improves pain and walking function in geriatric knee osteoarthritis via proprioceptive mediation

**DOI:** 10.3389/fpain.2026.1749582

**Published:** 2026-02-26

**Authors:** Bowen Zhu, Sipeng Huang, Shuaipan Zhang, Qingguang Zhu, Min Fang

**Affiliations:** 1 Institute of Manual Therapy, Yueyang Hospital of Integrated Traditional Chinese and Western Medicine, Shanghai University of Traditional Chinese Medicine, Shanghai, China; 2 Department of Traditional Chinese Massage, Yueyang Hospital of Integrated Traditional Chinese and Western Medicine, Shanghai University of Traditional Chinese Medicine, Shanghai, China; 3 Department of Traditional Chinese Massage, Shuguang Hospital, Shanghai University of Traditional Chinese Medicine, Shanghai, China

**Keywords:** force sense, knee osteoarthritis (KOA), manual therapy (MT), pain, proprioceptive, walking function

## Abstract

**Objective:**

To evaluate the therapeutic effects of manual therapy (MT) on pain, walking function, proprioception, and lower-limb muscle strength in geriatric patients with knee osteoarthritis (KOA), and to explore potential biomechanical mechanisms.

**Methods:**

In this prospective non-randomized controlled trial, 25 geriatric participants with unilateral mild-to-moderate KOA (intervention group) and 25 matched healthy controls were enrolled. The intervention group underwent a 4-week MT, while the control group received no intervention. The primary outcome was change in Western Ontario and McMaster Universities osteoarthritis index (WOMAC) pain subscale score. Secondary outcomes included 6-minute walk test (6-MWT) distance, proprioceptive force sense (PFS), and maximal isometric lower-limb strength. Causal mediation analysis was employed to investigate mechanistic pathways linking pain modulation to functional recovery.

**Results:**

Following MT intervention, the intervention group demonstrated significant reductions in WOMAC pain scores (13.04 ± 4.39 vs. 5.36 ± 2.40; *P* < 0.001), increased 6-MWT distance [320.44 m [95% CI: 283.68–357.20] vs. 612.28 m [95% CI: 594.42–630.14]; *P* < 0.001], and diminished inter limb asymmetry in PFS (54.52 ± 19.35 N vs. 15.92 ± 11.00 N; *P* < 0.05). Mediation analysis revealed that restoration of proprioceptive sensitivity accounted for 69.59% of the total effect through which pain reduction enhanced ambulatory capacity. Muscle strength profiling further indicated that functional improvement was potentially mediated by neuromuscular rebalancing of bilateral agonist-antagonist coordination.

**Conclusion:**

MT delivers significant short-term clinical benefits for elderly patients with unilateral mild-to-moderate KOA (Kellgren-Lawrence grades 0–2), including effective pain relief and improved walking ability, potentially through restoring proprioceptive sensitivity and modulating intermuscular balance.

## Introduction

Knee osteoarthritis (KOA) affects an estimated 654 million people globally, with a prevalence of 16.0% and an annual incidence of 2.03% (1, 2). As one of the most common degenerative knee diseases, the senior population is at particularly high risk for KOA (3) and the negative effects of KOA (knee pain, limited mobility, and proprioceptive dysfunction, etc.) significantly affect patients' quality of life (4, 5).

Proprioceptive deficits which are frequently overlooked in routine rehabilitation assessments, are common in older adults with KOA, and these deficits arise from structural factors such as muscle atrophy and neural degeneration (6). This somatosensory dysfunction severely compromises patients' balance, increases fall risk, and may indirectly contribute to secondary disability and accidental injury (7). Pain induced by knee movement further impairs central control over the affected knee, creating a vicious cycle of “pain-induced sensory impairment” (8).

Manual therapy (MT) may exert analgesic effects in patients with KOA through synergistic regulation across multiple brain regions, including the thalamus, postcentral gyrus, and insula (9, 10). However, clinical studies have rarely investigated the impact of MT on proprioceptive recovery in KOA patients. Based on this, we hypothesized that MT's regulatory mechanism for central analgesia in KOA patients may concurrently enhance somatic proprioceptive function. In this study, we demonstrated the benefits of MT on pain, mobility, and proprioceptive sensations in patients with KOA by comparing them with normal participants, and observed changes in isometric contraction muscle strength in KOA after MT.

## Methods

### Study design

We conducted a prospective, non-randomized controlled trial comparing clinical and biomechanical outcomes before and after a 1-month MT intervention in patients with KOA. The study design was approved by the Ethics Committee of Shuguang Hospital, Shanghai University of Traditional Chinese Medicine (Approval NO. 2023-1357-124-01) and conducted in accordance with the Declaration of Helsinki. Written informed consent was obtained from all participants.

### Participants

The study was conducted at the Department of Tuina, Shuguang Hospital Affiliated with Shanghai University of Traditional Chinese Medicine, China. Participants were recruited through posters, clinical referrals, and a WeChat application. Recruitment commenced in August 2023 and was completed in March 2025. The study employed a pairwise matching strategy. Potential participants were initially contacted by phone by the study coordinator to ascertain their willingness to participate. Those who met the diagnostic criteria for KOA (11) and the inclusion criteria (Table 1) were invited to the biomechanics laboratory for baseline assessments. Subjective scale scores, objective physical function data, and biomechanical data were recorded. Subsequently, basic information [gender, age, Body Mass Index (BMI)] for each participant was recorded on an envelope.

**Table 1:** Eligibility criteria.

Inclusion criteria for KOA:	Exclusion criteria for KOA:
Unilateral KOA with contralateral knee clinically normal	Recent knee interventions (physiotherapy or intra-articular injections for KOA pain within 1 month)
Aged >50 and <80 years	Significant comorbidities: - Major systemic diseases (e.g., NYHA Class III/IV heart failure, ESRD, uncontrolled diabetes) - Concurrent chronic pain disorders (e.g., fibromyalgia, neuropathic pain)
Mild-to-moderate KOA severity (defined as total WOMAC score <48 points)	Communication barriers affecting protocol compliance
KL grade 0 to II on radiographic assessment^#^	Positive ballottement test (indicating joint effusion) or Peripatellar skin lesions or infections
No therapeutic interventions for KOA-related pain (including pharmacotherapy) within 7 days prior to enrollment	Current participation in other clinical trials
Voluntary participation with written informed consent	

KOA, Knee osteoarthritis; NYHA, New York Heart Association; ESRD, End-Stage Renal Disease; KL, Kellgren-Lawrence; WOMAC, Western Ontario and McMaster Universities osteoarthritis index. #Grading 0, No radiographic abnormalities and absence of osteophytes or joint space narrowing; Grading I, Possible minute osteophytes and questionable joint space narrowing; Grading II: Definite osteophytes and possible joint space narrowing.

### Subgroups and sample size calculation

We employed a case-control design based on baseline characteristics. All eligible KOA patients were first identified as candidate cases. Subsequently, using restrictive matching, a healthy individual most closely matched to each intervention group case in terms of baseline variables such as sex, age, and BMI was selected from the same study cohort to serve as a control. The sample size was determined based on previous exploratory studies (*n* = 20 per group) that detected significant between-group differences in similar contexts (12). To ensure statistical power and allow for potential dropouts, the sample size was increased by 25% to 25 participants per group, resulting in a total target sample size of 50 participants. Sample size calculation using G*Power 3.1 software (*α* = 0.05, two-tailed; *β* = 0.20) confirmed that a sample size of 25 participants per group provided statistical power exceeding 80% for detecting an effect size (Cohen's *d*) ≥ 0.7 (moderate-to-large). This effect size threshold aligns with effects observed in prior studies (12) and reflects the assumption that clinically meaningful differences would be detectable if present, balancing feasibility with statistical power.

### Intervention

Participants in the control group received no therapeutic intervention and were instructed to maintain their usual daily routines while avoiding structured lower extremity strength training and any sports or activities posing a risk of knee injury.

Participants in the intervention group underwent a detailed physical examination and comprehensive health education at the first visit, and relevant medical counseling services were provided to the participants as much as possible if they needed them (Supplementary Appendix 1). The intervention group received MT twice weekly, with each session lasting approximately 20 min, for a total of 4 consecutive weeks. Manual therapists, each with over 5 years of professional experience, received standardized training on the trial protocol, diagnostic criteria, and treatment procedures for KOA prior to study initiation to minimize inter-therapist variability. A standardized check-in procedure was implemented for each MT session, and session attendance was recorded in an Excel spreadsheet. Participants scheduled their treatment appointments for the following week at the end of each preceding week. At the end of each week, participants made appointments for the following week, so that the treating physicians tried to ensure that each participant was treated at the same time each week. MT technology was based on the relevant operations in the published randomized controlled trial for the treatment of KOA (13), including relaxing the soft muscle group tissues of the affected limb, stimulating trigger points to adjust ligament activity, thumb pressing to activate muscle activity, and gentle auxiliary passive joint movement of the knee joint. The detailed treatment protocol is provided in Supplementary Appendix 2.

Participants with KOA were instructed to avoid any treatment for KOA during these period concomitant KOA treatments (e.g., analgesics, glucocorticoids, physical therapies). Should the patient experience intolerable pain and remain unassessed within 48 h, analgesic medication may be administered. At the first visit, participants provided information on any other medical conditions they currently had and the appropriate medications they were using, all of which were be recorded on the Case Report Form (CRF)form and all treatments were documented, including the name of the medication dosage and duration of the treatment, and the time of the start and end of the administration of the medication.

### Outcome assessments

Outcomes were assessed at baseline and after 4 weeks of treatment encompassing subjective scale scores, objective physical function data, and biomechanical data. To ensure confidentiality, no personal information pertaining to potential participants or registered participants that was unrelated to the trial was collected, shared, or stored at any stage. Data processing was performed in an anonym zed manner. Outcome assessors and statisticians were blinded to group allocation. However, due to the nature of the intervention, participants and treating therapists could not be blinded.

#### Primary outcome

##### Western Ontario and McMaster Universities osteoarthritis index (WOMAC) pain score

The WOMAC pain subscale score was used to assess changes in pain intensity among KOA patients before and after treatment (14). The WOMAC pain score consisted of 5 items to evaluate the pain of participants in the state of walking, going up and down stairs, sleeping, sitting or lying down, and standing, with a total score of 20 points, with higher scores indicating more severe osteoarthritis. If a participant had difficulty understanding the evaluation content, they completed the form with assistance from a neutral third party to ensure the accuracy of the evaluation results.

#### Secondary outcomes

##### 6-minute walk test (6-MWT)

The 6-MWT was used to assess the walking capacity and exercise tolerance of the patients during their daily activity state (15). The test was conducted indoors on a long, straight, flat, 30-meter walking course in a minimally trafficked corridor. Participants were instructed to walk at their habitual, comfortable pace (no running or jogging) and were permitted to slow down or rest if necessary during the test. The total distance covered in 6 min was recorded.

##### Proprioceptive force sense (PFS) testing

PFS was assessed using the DIERS myoline professional system (Germany, DICAM version 2.0.0) to evaluate knee joint proprioception in both lower limbs (16). Testing procedure: (1) Participants sat on the testing chair with the torso secured by straps. The hips and knees were positioned at 90° flexion (thighs horizontal, calves perpendicular to thighs). Resistance pads were fixed 2 cm proximal to the medial malleolus. Participants wore blindfolds, and the room was kept quiet. (2) Participants were instructed to release equal forces from both knees at the same time and hold them for at least 5 s within the maximum force limit. (3) The magnitude of force generated during knee extension, adduction, and abduction was recorded separately for the healthy and affected limbs. The absolute difference in force sense between limbs was calculated and defined as the PFS asymmetry.

##### Maximal isometric lower-limb muscle strength

Maximal isometric muscle strength of the lower limbs was assessed using the DIERS myoline professional system (Germany, DICAM version 2.0.0) (17). During the test, the participant was measured with the pelvis resting as far as possible against the seat back and the belt was tightened; the participant's leg was tested with the leg placed on the swivel arm, the calf between the two cushioning rings, the hip adducted by removing the adducted support cushion, and the back shoulder cushion was adjusted to the base of the deltoid muscle. Participants were instructed to release the maximum force of both lower limbs at the same time, and perform knee joint adduction, abduction, extension and flexion in sequence, maintaining each movement at the end of the movement for at least 5 s, and resting for 1–2 min between movements until the patient's self-perceived strength was fully restored. The isometric contraction strength of both lower limbs was collected, and the difference between the healthy and affected sides/left and right sides of the participant was taken to assess the improvement of isometric contraction strength of the knee joint.

### Safety evaluation

Any adverse events that occurred during the treatment period were reported to the investigator for recording and this record was statistically recorded on a weekly basis.

### Patient and public involvement

Patients or the public were not involved in the design, conduct, reporting, or dissemination plans of this research.

### Statistical methods

Baseline data, including demographic characteristics, disease-related indicators, and other institutional data, were recorded in Microsoft Excel 2019. Statistical analyses were performed using IBM SPSS Statistics software (version 27.0). Missing data were imputed in using stochastic imputation. The minimum clinically important difference (MCID) threshold for the WOMAC pain subscale was defined as an improvement of 2.12 points (representing a 20% change from baseline), based on previous studies (18).

Categorical variables were analyzed using the chi-square (*χ*^2^) test or Fisher's exact test. Continuous variables conforming to a normal distribution were analyzed using the independent samples *t*-test, while the Mann–Whitney *U*-test was used for non-normally distributed continuous variables. Measurements were expressed as mean Mean ± SD or Mean, 95% CI, and count data were expressed as frequency (percentage, *n* [%]). The Shapiro–Wilk test was used to assess normality, and Levene's test was used to assess homogeneity of variance for all outcome measures. For data that were normally distributed and variance-aligned, the paired samples t-test was used for within-group comparisons (before and after treatment), and the independent samples *t*-test was used for between-group comparisons (endpoints or improvement values). For data violating normality or homogeneity of variance assumptions, the Wilcoxon signed-rank test was used for within-group comparisons, and the Mann–Whitney *U*-test was used for between-group comparisons.

Mediation analysis within the intervention group was conducted using the PROCESS macro (version 3.5) for SPSS.A mediation model (X → M → Y) was constructed with the value of improvement in WOMAC pain score as the independent variable (X), the value of improvement in proprioception as the mediating variable (M), and the value of improvement in 6-minute walking distance as the dependent variable (Y), and the significance of the indirect effect was tested using Bootstrap method to explore the possible mechanisms of improvement in walking ability (6-MWT).

## Results

### Baseline characteristics of patients

**Figure 1:**
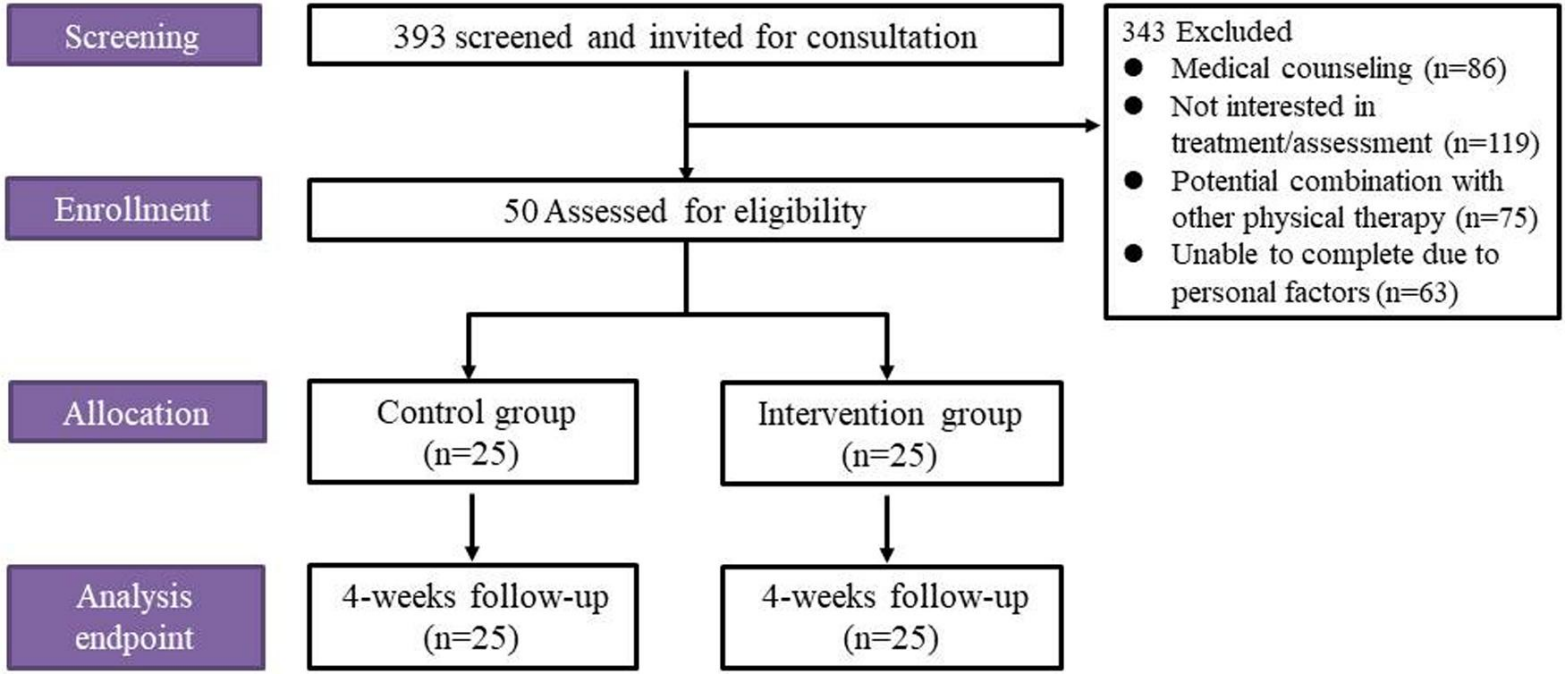
Flow chart of study participants.

Between August 2023 and March 2025, 393 potential participants were screened. After excluding those not meeting all inclusion criteria, 50 participants were enrolled. All participants completed the 4-week assessment (Figure 1). We conducted a statistical analysis of the educational attainment of the subjects to control for the possible confounding bias it might bring to the results. Baseline characteristics of the participants are presented in Table 2.

**Table 2:** Patient characteristics.

Characteristic	Intervention group (*n* = 25)	Control group (*n* = 25)	*t*, *χ*^2^, *Z*-value	*P* value
Age (years)	59.52 ± 8.30	60.68 ± 8.74	−0.481	0.633
Sex				
Female (*n*, %)	16 (64%)	16 (64%)		
Male (*n*, %)	9 (36%)	9 (36%)		
BMl (kg/m^2^)	25.94 ± 1.76	24.84 ± 2.28	1.908	0.062
Degree of education				
Junior high school and below (*n*, %)	7 (28%)	6 (24%)		
High school (*n*, %)	13 (52%)	15 (60%)		
Bachelor's degree or above (*n*, %)	5 (20%)	4 (16%)		
Disease course (years)	6.31 ± 2.31	–		
Affected limb		–		
Left side (*n*, %)	12 (48%)			
Right side (*n*, %)	13 (52%)			
KL grade		–		
Grade I (*n*, %)	6 (24%)			
Grade II (*n*, %)	19 (76%)			
WOMAC pain (score)	13.04 ± 4.39	–		
6-MWT (m)	320.44 (283.68, 357.20)	609.40 (585.87, 632.93)	−6.063	<0.001

BMI, Body Mass Index; KL, Kellgren-Lawrence; WOMAC, Western Ontario and McMaster Universities osteoarthritis index; 6-MWT, 6-minute walk test.

### Primary outcomes

WOMAC pain scores significantly decreased from baseline to post-treatment (13.04 ± 4.39 vs. 5.36 ± 2.40; *P* < 0.001), exceeding the predefined MCID threshold. Detailed result values were in Table 3.

**Table 3:** Pain, walking functional capacity, and PFS outcomes.

Item		Intervention phase	Intervention group (*n* = 25)					Control group (*n* = 25)					Inter-group comparison	
Pain			Value	*t*, Z-value		*P* value		Value	*t*, *Z*-value		*P* value		*t*, *Z*-value	*P* value
WOMAC pain (score)		Baseline	13.04 ± 4.39	7.238		<0.001		–						
		4 weeks	5.36 ± 2.40											
Walking Functional Capacity														
6-MWT (m)		baseline	320.44 (283.68, 357.20)	−4.373		<0.001		609.40 (585.87, 632.93)	−0.942		0.346		−6.063	<0.001
		4 weeks	612.28 (594.42, 630.14)					619.72 (599.20, 640.24)					−0.747	0.455
**PFS**				**Hea and aff sides Comparison**		**Pre - and post-treatment Comparison**			**L and R sides Comparison**		**Baseline and 4weeks Comparison**			
Detection of lower limb proprioceptive strength				***t*-value**	***P* value**	***t*-value**	***P* value**		***t*-value**	***P* value**	***t*-value**	***P* value**		
**Adduction (*N*)**	Hea/L	Baseline	1,078.00 ± 157.93	3.021	0.004			1,263.44 ± 175.44	−0.182	0.856				
	Aff/R		946.32 ± 150.18					1,272.72 ± 184.14						
		|d|	138.96 ± 68.35					32.00 ± 20.02					7.509	<0.001
	Hea/L	4 weeks	1,040.60 ± 176.41	1.552	0.127	0.788	0.438	1,315.56 ± 188.63	−0.093	0.926	−1.022	0.317		
	Aff/R		963.60 ± 174.34			−0.362	0.720	1,320.52 ± 187.13			−0.910	0.372		
		|d|	95.40 ± 52.02			2.627	0.015	39.76 ± 17.85			−1.330	0.196	5.058	<0.001
**Abduction (*N*)**	Hea/L	Baseline	874.32 ± 73.18	−1.441	0.162			869.80 ± 66.71	0.188	0.851				
	Aff/R		883.76 ± 70.31					865.92 ± 78.55						
		|d|	137.52 ± 94.92					27.40 ± 20.21					5.674	<0.001
	Hea/L	4 weeks	960.08 ± 94.42	0.728	0.474	−3.590	0.001	867.40 ± 76.48	−0.512	0.611	0.333	0.742		
	Aff/R		939.48 ± 90.06			−2.438	0.019	878.76 ± 80.51			−1.521	0.141		
		|d|	73.96 ± 52.74			3.521	0.002	29.76 ± 20.56			−0.380	0.707	3.904	<0.001
**Extension (*N*)**	Hea/L	Baseline	115.48 ± 20.13	12.222	<0.001			154.40 ± 50.55	−0.251	0.803				
	Aff/R		60.96 ± 9.61					158.04 ± 51.99						
		|d|	54.52 ± 19.35					9.80 ± 7.59					−9.331	<0.001
	Hea/L	4 weeks	77.48 ± 15.81	3.952	<0.001	12.827	<0.001	156.40 ± 43.80	−0.234	0.816	−0.708	0.486		
	Aff/R		61.56 ± 12.46			−0.462	0.648	159.48 ± 48.97			−0.644	0.526		
		|d|	15.92 ± 11.00			14.813	<0.001	10.04 ± 7.96			−0.114	0.910	2.166	0.035

WOMAC, Western Ontario and McMaster Universities osteoarthritis index; 6-MWT, 6-minute walk test; PFS, proprioceptive force sense; Hea, Health limb; Aff, Affected limb; L, left; R, Right; |d|, The absolute inter limb difference in proprioceptive strength between the Aff and Hea limbs, or between the L and R limbs.

### Secondary outcomes

- 6-MWT results

The 6-MWT distance significantly increased in the intervention group from baseline to post-treatment [320.44 m, 95% CI [283.68, 357.20] vs. 612.28 m, 95% CI [594.42, 630.14]; *P* < 0.001]. At the 4-week assessment, there was no significant difference in 6-MWT distance between the intervention group and the control group [612.28 m, 95% CI [594.42, 630.14] vs. 619.72 m, 95% CI [599.20, 640.24]; *P* = 0.346]. Detailed result values were in Table 3.

- PFS results

PFS results revealed significant baseline asymmetry between limbs in KOA patients. PFS during adduction and extension was higher in the healthy limb compared to the affected limb (adduction: 1,078.00 ± 157.93 N vs. 946.32 ± 150.18 N, *P* = 0.004; extension: 115.48 ± 20.13 N vs. 60.96 ± 9.61 N, *P* < 0.001). After 4 weeks of MT, PFS during adduction did not differ significantly between the healthy and affected limbs (1,040.60 ± 176.41 N vs. 963.60 ± 174.34 N; *P* = 0.127). However, PFS during extension remained significantly higher in the healthy limb compared to the affected limb (77.48 ± 15.81 N vs. 61.56 ± 12.46 N; *P* < 0.001). While significant inter-limb differences in PFS persisted across activities in the intervention group post-treatment compared to the control group (all *P* < 0.05), the magnitude of this inter-limb asymmetry was significantly reduced within the intervention group compared to baseline for all tested movements (all *P* < 0.05). Detailed result values were in Table 3.

- Maximal isometric lower-limb muscle strength results
  - Adduction and abduction

Before treatment, patients with KOA demonstrated weaker maximal adductor strength on the affected side than on the healthy side (1,086.20 ± 146.45 vs. 1,306.88 ± 198.49, *P* < 0.001). After treatment, the difference between the intrinsic muscle strength of the healthy and affected sides of the KOA decreased (220.68 ± 150.95 vs. 95.96 ± 130.77, *P* = 0.003). Adductor strength on the affected side of the KOA showed a significant decrease from before to after treatment (1,139.32 ± 190.23 vs. 1,074.28 ± 140.47, *P* = 0.034) and the inter-limb difference in adductor strength increased significantly from baseline to post-treatment (−45.24 ± 143.16 vs. 36.08 ± 181.87, *P* = 0.019).

- Flexion and extension

Before treatment, patients with KOA demonstrated weaker flexor strength on the affected side than on the healthy side (38.60 ± 14.89 vs. 64.52 ± 14.62, *P* < 0.001). After treatment, there was no significant change in flexor strength on the affected side of the KOA compared with the previous side, and flexor strength on the healthy side was significantly reduced compared with the pre-treatment side (48.00 ± 13.70 vs. 64.52 ± 14.62, *P* = 0.041). Before treatment, patients with KOA demonstrated weaker extension muscle strength on the affected side than on the healthy side (241.84 ± 39.00 vs. 321.64 ± 43.30, *P* < 0.001), i.e., the affected side presented weaker quadriceps strength. After treatment, the patient's extensor strength was significantly reduced bilaterally compared to before (288.16 ± 41.28 vs. 321.64 ± 43.30, *P* < 0.001; 225.44 ± 37.06 vs. 241.84 ± 39.00, *P* < 0.001).

Detailed result values were in Table 4.

- Mediation analysis results

Mediation analysis revealed that the total effect was *β* = 5.13 and the indirect effect mediated by improvement in extension PFS was significant (*β* = 3.57, bootstrap 95% CI: 0.13 to 7.31). The proportion of the total effect mediated by improvement in extension PFS was 69.59% (indirect effect *β* = 3.57/total effect *β* = 5.13), indicating that this pathway was the primary mechanism through which pain reduction enhanced walking capacity (Tables 5, 6).

**Table 4:** Maximal isometric lower-limb muscle strength results.

Isometric contraction muscle strength		Intervention phase	Intervention group (*n* = 25)					Control group (*n* = 25)					Inter-group comparison	
			Value	Hea and Aff sides Comparison		Pre- and post-treatment Comparison		Value	L and R sides Comparison		Baseline and 4weeks Comparison		*t*-value	*P* value
				*t*-value	*P* value	*t*-value	*P* value		*t*-value	*P* value	*t*-value	*P* value		
**Adduction (*N*)**	Hea/L	Baseline	1,306.88 ± 198.49	4.473	<0.001			1,320.12 ± 175.76	−0.223	0.825				
	Aff/R		1,086.20 ± 146.45					1,331.00 ± 169.32						
		d	220.68 ± 150.95					10.88 ± 113.35					5.557	<0.001
	Hea/L	4 weeks	1,198.56 ± 132.49	3.508	0.001	2.581	0.016	1,322.40 ± 172.19	−0.211	0.833	−0.076	0.940		
	Aff/R		1,126.60 ± 112.45			−1.093	0.285	1,332.24 ± 156.53			−0.071	0.944		
		d	95.96 ± 130.77			3.123	0.003	9.84 ± 142.96			0.029	0.977	2.222	0.031
**Abduction (*N*)**	Hea/L	Baseline	1,094.08 ± 182.37	−0.858	0.395			1,382.36 ± 216.80	0.193	0.847				
	Aff/R		1,139.32 ± 190.23					1,369.12 ± 264.91						
		d	−45.24 ± 143.16					−13.24 ± 135.97					−0.810	0.422
	Hea/L	4 weeks	1,110.36 ± 188.48	0.767	0.447	−1.365	0.185	1,350.44 ± 219.16	0.275	0.784	1.968	0.061		
	Aff/R		1,074.28 ± 140.47			2.244	0.034	1,332.36 ± 244.39			1.870	0.074		
		d	36.08 ± 181.87			−2.525	0.019	−18.08 ± 145.77			0.171	0.866	1.162	0.251
**Extension (*N*)**	Hea/L	Baseline	321.64 ± 43.30	6.847	<0.001			325.60 ± 53.79	−0.323	0.748				
	Aff/R		241.84 ± 39.00					330.44 ± 52.16						
		d	79.80 ± 34.64					4.84 ± 61.51					5.309	<0.001
	Hea/L	4 weeks	288.16 ± 41.28	5.654	<0.001	6.730	<0.001	329.00 ± 49.16	0.160	0.874	−0.306	0.762		
	Aff/R		225.44 ± 37.06			3.079	<0.001	326.64 ± 55.14			0.490	0.629		
		d	62.72 ± 27.48			2.310	0.030	−2.36 ± 56.15			0.804	0.429	5.205	<0.001
**Flexion (*N*)**	Hea/L	Baseline	64.52 ± 14.62	6.210	<0.001			78.12 ± 18.87						
	Aff/R		38.60 ± 14.89					80.88 ± 18.11						
		d	25.92 ± 10.25					2.76 ± 23.79					4.470	<0.001
	Hea/L	4 weeks	48.00 ± 13.70	2.096	0.041	9.177	<0.001	81.48 ± 17.28	−0.345	0.731	−1.686	0.105		
	Aff/R		39.68 ± 14.36			−1.538	0.137	83.12 ± 16.27			−1.138	0.267		
		d	8.32 ± 5.94			8.852	<0.001	1.64 ± 25.54					1.494	0.142

Hea, Health limb; Aff, Affected limb; L, left; R, Right; d, The difference in proprioceptive strength sensation between the affected and healthy limbs or between the left and right limbs.

**Table 5:** Correlations between change scores (X, M, Y).

Factors	Mean	SD	X	M	Y
X	7.68	5.31	1		
M	38.60	13.03	0.612^a^	1	
Y	288.96	60.76	0.448^b^	0.592^c^	1

X, Western Ontario and McMaster Universities osteoarthritis index (WOMAC) pain improvement value; Y, 6-minute walk test (6-MWT) improvement value; M, Improvement in extension of PFS.

a *P* = 0.001.

b *P* = 0.025.

c *P* = 0.002.

**Table 6:** Mediation analysis: decomposition of effects.

Effects	PE	Se	LLCI	ULCI	ES
Te	5.13	2.14	0.72	9.55	100%
De	1.56	2.47	−3.55	6.68	30.41%
Me	3.57	1.82	0.13	7.31	69.59%

PE, Point Estimate; Se, Standard Error; LLCI, Lower Limit of Confidence Interval; ULCI, Upper Limit of Confidence Interval; ES, Effect Size; Te, Total effect; De, Direct effect; Me, Mediating effect.

### Adverse events

No serious adverse events occurred. One female participant in the intervention group experienced hypoglycemic symptoms following muscle strength testing performed in a fasting state. Symptoms resolved immediately with supine positioning, leg elevation, and oral glucose administration. No recurrence was reported during a 1-month telephone follow-up.

## Discussion

For chronic KOA, long-term management is paramount. However, KOA patients typically presented characteristics such as advanced age, lower limb muscle atrophy, and cognitive impairment, which constrained the long-term feasibility and implementation effectiveness of guideline-recommended conservative treatments (e.g., weight reduction and quadriceps strengthening exercises), leading to persistent management challenges (19, 20). Particularly in resource-constrained settings, elderly patients often struggled to access sustained private guidance from physiotherapists. Concurrently, activity limitations arising from KOA further diminish patient adherence to active exercise therapy. Patient compliance is a prerequisite for therapeutic efficacy; therefore, passive treatment strategies such as MT may hold potential value within KOA management protocols, especially when patient adherence to active exercise is limited.

MT has a positive impact on alleviating pain, negative emotions and disability in patients with KOA (13). Our previous meta-analysis similarly confirmed that, in the short term, MT may offer analgesic benefits comparable to exercise therapy, but the pain-relieving effects may be limited in certain specific activities associated with KOA (21). Concerns persist among many patients and clinicians regarding the effectiveness and underlying mechanisms of MT (22). Therefore, investigating the relationship between MT and its effects on pain relief and functional capacity in KOA warrants particular attention.

Our study demonstrated that 4 weeks of MT improved pain, walking ability, and proprioception and observed changes in the balance of isometric muscle strength in both lower limbs before and after treatment in unilateral mild-to-moderate geriatric KOA (Kellgren-Lawrence grades 0–2). MT may mediate pain relief and enhanced walking ability through the restoration of proprioceptive force perception, but as our research was a non-randomized controlled trial with a relatively brief intervention period, this inference was likely subject to limitations arising from causal bias in the outcomes.

The research results showed that (i) compared with pre-treatment levels, the intervention group exhibited a significant reduction in WOMAC pain scores after 4 weeks of treatment (*P* < 0.001); (ii) the 6-MWT demonstrated a significant improvement compared with pre-treatment levels (*P* < 0.001) and was comparable to the control group (*P* = 0.455). Although differences in PFS persisted between the intervention group and the control group following treatment (both *P* < 0.05), compared with pre-treatment levels, PFS differences in both knees significantly narrowed (both *P* < 0.05) in the intervention group, which made the force generation pattern driving bilateral lower limb closer, and MT might have positive effects on the improvement of the PFS in KOA with a positive driving effect. (iii) Mediation analysis resulted indicated that improvements in patients' extension PFS ability played moderate mediating role in pain relief and gait improvement, with an indirect effect size of 69.59%. We deduced a hypothesis-generating finding that individuals experiencing more pronounced improvements in WOMAC pain scores might have achieved greater gains in 6-MWT distance through enhanced proprioceptive sensitivity in the knee extensor muscles and MT might enhance knee extension PFS in geriatric KOA by alleviating pain, thereby promoting improvements in walking ability.

Unlike guideline-recommended quadriceps strengthening exercises, MT, as a passive modality, did not directly increase lower-limb muscle strength. Increased muscle mass typically corresponds with a greater density of proprioceptors; thus, quadriceps strengthening contributes to proprioceptive restoration in KOA (23). To explore potential mechanisms underlying MT's effects on proprioceptive recovery in KOA, we assessed changes in maximal isometric lower-limb muscle strength. Contrary to exercise therapy which directly enhanced muscle strength and found that following MT treatment, strength in the unaffected limb had decreased compared to previous levels. However, it remained uncertain whether these observed changes in muscle strength should be regarded solely as alterations accompanying MT treatment, as our study couldn't rigorously isolate the specific effects of MT. These improvements are unlikely to be explained solely by temporal factors, as placebo effects, regression to the mean, learning effects from repeated isometric testing, or natural symptom fluctuations could also influence muscle strength measurements in KOA patients.

Our findings aligned with previous observations that patients with KOA might exhibit sufficient net quadriceps force partly due to co-activation of antagonist muscles (e.g., hamstrings), which could paradoxically limit force output in the affected limb (24). However, this equilibrium stabilization was also flawed in the sense that it was a sacrifice of some of the healthy side's muscle strength to maintain the relative equilibrium of the lower limb strength, which might go some way towards explaining why hyperkinesia in patients with KOA tended to be accompanied by a bilaterally sequential appearance (25). Whether MT can generate a neuron-muscular protective adaptation mechanism by reducing force output in healthy limbs, thereby effectively alleviating bilateral muscle imbalance during exertion to maintain movement equilibrium and stability, remains to be further explored. These findings supported MT's clinical utility while highlighting the need to balance-focused strengthening exercises from the initial treatment phase.

## Limitations

First, during the development of the study protocol, the effect of MT on proprioception in KOA had not been previously reported, making it difficult to predict the effect size of the effects of MT on proprioception in KOA. Therefore, this study was only designed as an exploratory prospective non-randomized controlled study with a small sample size that limited the quality level of the evidence for the research results. Secondly, KOA with single knee and graded Kellgren-Lawrence grades 0–2 involvement included in the study only, and the results might not be generalizable to patients with two-knee lesions of KOA or to patients with more severe KOA. Finally, the limitation of our proprioceptive testing protocol lies in its failure to assess proprioceptive force perception capabilities in the direction of knee flexion. This arises from the inherent constrained of the seated testing posture used to measure knee extension force, which restricted the generation of force in the posterior direction of the knee joint. Consequently, proprioceptive data pertaining to flexion cannot be obtained.

## Conclusion

MT may yield significant short-term clinical benefits for elderly patients with unilateral mild-to-moderate knee osteoarthritis (Kellgren-Lawrence grades 0–2), including effective pain relief, improved walking ability, and positive effects on promoting proprioceptive sensitivity. The potential mechanism may involve promoting beneficial improvements in proprioceptive sensitivity and bilateral lower limb muscle strength balance. However, MT is not recommended as a long-term standalone management strategy. The observed reduction in maximal strength of the contralateral limb suggests a potential compensatory adaptation that warrants caution and further investigation regarding long-term use.

## Data Availability

The raw data supporting the conclusions of this article will be made available by the authors, without undue reservation.
