# Supplementary material for: Manual therapy improves pain and walking function in geriatric knee osteoarthritis via proprioceptive mediation
Source: Front Pain Res (Lausanne). 2026 Feb 26;7:1749582. doi: 10.3389/fpain.2026.1749582 (PMC12979429; doi:10.3389/fpain.2026.1749582)
Supplement: Supplementary file 1 [file Table1.docx]

**Appendix**

**Appendix 1 Content of Health Education and Medical Consultation**

**1. Health Education**

The main content of health education includes helping the subjects to change the bad lifestyle, effective weight control and encouraging the lower limb functional exercises suitable for themselves.

Specific content: (i) Advise KOA patients to maintain normal body mass, avoid progressive weight gain, and control weight to reduce the load on the knee joint; (ii) Pay attention to the local joint warmth of the knee joint, avoid wind, cold and dampness, and avoid trauma to the knee joint; (iii) Do not overstretch the ligaments, tendons, and the joint itself during knee joint activities; (iv) Avoid prolonged repetitive movements or maintaining the joint posture for a long period of time, and try to avoid or reduce the tempo of Avoid doing fast or unstoppable movements, and brake immediately if knee pain occurs; (v) Ask female KOA to reduce the use of high-heeled shoes; (vi) Ask the patient to reduce walking and increase the sitting time during the acute attack of KOA, and avoid premature functional exercise during the inflammation period, and then perform appropriate exercise with less impact on the joints after the inflammation has subsided.

**2. Medical counseling**

Medical counseling services help them understand the disease and its treatment goals, and encourage them to make lifestyle changes and adopt behaviors that may be beneficial to their condition or at least prevent it from worsening.

The main framework of the service includes: (i) Explaining the diagnosis and possible causes of KOA, as well as helping to deal with patients' questions about KOA; (ii) Informing them of the importance of lifestyle interventions, such as regular physical activity, nutrition, sleep and stress management;(iii) Educating them about unexpected high-risk events;(iv) Explaining issues related to joint loading and the dosage of physical activity/exercise, etc.; (v) Advising them to control their body weight and to maintain an average body weight. Advise patients with normal weight on the importance of maintaining a normal weight range; (vi) Evaluating patients who are obese or overweight and assist with weight loss advice and weight loss programs, etc.

**Appendix 2 Operative protocols for manual treatment(MT) of KOA**

The specific program of MT is as follows: (i) Relaxation of muscles. With the patient in the prone position and the practitioner standing/sitting on the patient's affected side, first relax the affected semitendinosus muscle and biceps femoris muscle; then instruct the patient to lie supine and relax the lateral femoral muscle for a total of about 5 min. (ii) Stimulation of pain points. With the patient lying in the supine position, the doctor used the thumb to explore the pain points with strips around the knee, and then used the thumb to flick the pain points and stimulate the inner and outer collateral ligaments of the lower limbs for 8-10 min; (iii) Activating muscle groups. The doctor uses the thumb to press the rectus femoris muscle, medial femoris muscle and gastrocnemius muscle belly on the affected side to the extent that the patient feels soreness and swelling, and presses each point for about 1 min; (iv) Move the joints. The healer presses the palm against the knee circumference, warms the knee circumference clockwise, and then shakes the knee joint for 3-5 times.
